# Ibrutinib dose modifications in the management of CLL

**DOI:** 10.1186/s13045-020-00870-w

**Published:** 2020-06-05

**Authors:** Camille Hardy-Abeloos, Rachel Pinotti, Janice Gabrilove

**Affiliations:** 1 Division of Hematology and Medical Oncology, Tisch Cancer Institute, Icahn School of Medicine at Mount Sinai, New York, NY USA; 2 Library Education and Research Services, Icahn School of Medicine at Mount Sinai, New York, NY USA

**Keywords:** Ibrutinib, CLL, dosage, discontinuation

## Abstract

**Background:**

Ibrutinib is a Bruton tyrosine kinase inhibitor approved for the treatment of chronic lymphocytic leukemia (CLL) in 2014. Ibrutinib is often used to treat patients who are younger than the patients originally included in theclinical trials have additional unfavorable prognostic factors and suffer from additional comorbidities excluded from the original phase III trials. Our objective was to examine current clinical practices and their impact in this expanded population of CLL patients who often require adjustments in the standard prescribed dose and schedule of therapy.

**Materials and methods:**

An extensive review of the medical literature was conducted to establish the consensus on ibrutinib dose modifications in patients with CLL. Twenty-nine studies were reviewed including fourteen clinical trials and fifteen “real-world practice” studies.

**Results:**

The average discontinuation rate was similar between clinical trials and “real-world practice” studies though the reasons for discontinuation differed. CLL progression was a more common reason for discontinuation in clinical trial studies while toxicity was a more common reason for discontinuation in “real-world practice” studies. Some studies have suggested worse outcomes in patients requiring dose reductions in ibrutinib while others have shown no change in treatment efficacy in patients requiring dose reductions due to concomitant CYP medications or increased immunosuppression post-transplant.

**Conclusion:**

The impact of ibrutinib dose modifications on clinical outcome remains unclear. Patients on concomitant CYP3A inhibitors should be prescribed a lower dose than the standard 420 mg daily, in order to maintain comparable pharmacologic properties. Further research is required to establish definitive clinical practice guidelines.

## Introduction

In 2014, ibrutinib, a Bruton’s tyrosine kinase (BTK) inhibitor, at a dose of 420 mg po daily, was approved as second line treatment for chronic lymphocytic leukemia (CLL), based on the RESONATE landmark trial, leading to FDA approval for this indication. Soon thereafter, the RESONATE-2 trial demonstrated the efficacy of this drug, utilizing the same dose and schedule, as first line therapy of CLL. The RESONATE-2 trial excluded patients who were younger than 65 years of age and who had chromosome 17p13.1 deletion. Both trials excluded patients who had an Eastern Cooperative Oncology Group (ECOG) performance status more than 2, inadequate kidney function, significant neutropenia or thrombocytopenia; patients requiring Warfarin due to the increased risk of bleeding on ibrutinib; and patients requiring strong CYP3A4/5 inhibitors due to ibrutinib’s metabolism by the same enzyme. Of note, patients requiring moderate or mild CYP3A inhibitors were not excluded from participation in these respective studies.

Since then, the use of ibrutinib to treat chronic lymphoid leukemia (CLL) has evolved. Currently ibrutinib is often used to treat patients who are younger than the patients originally included in the RESONATE and RESONATE-2 trials, respectively, have additional unfavorable prognostic factors and suffer from additional comorbidities excluded from the original phase III trials of ibrutinib. This expanded population of CLL patients, who are candidates for treatment with ibrutinib, often require adjustments in the standard prescribed dose and schedule of therapy. The purpose of this review was to examine our current knowledge of dose modifications of ibrutinib (dose reductions and/or interruption[s]) in “real-world practice” for patients with CLL and its potential clinical impact, as published in the literature.

## Methods

We searched PubMed and Scopus for studies examining the real-world dose reduction and interruption of ibrutinib in patient with CLL. Only studies published in English within the last 5 years, since the RESONATE trial in 2014, were included.

**Fig. 1:**
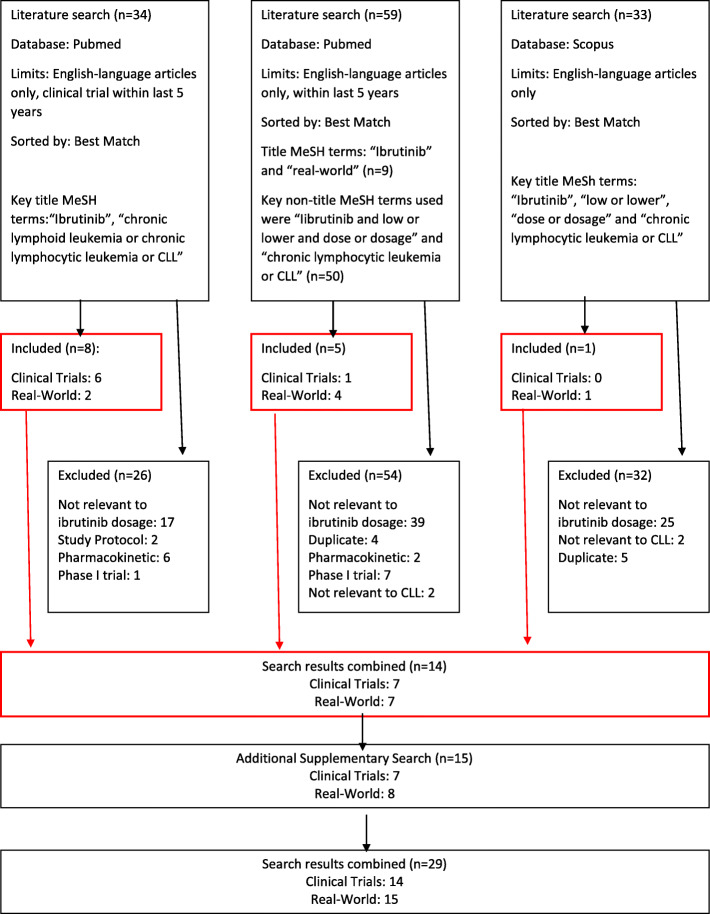
Flow Chart

To identify ibrutinib clinical trials in PubMed, the key title MeSH terms used were “Ibrutinib”, “chronic lymphoid leukemia or chronic lymphocytic leukemia or CLL” sorted by best match and filtered by clinical trial published in the last 5 years. Thirty-four papers were identified, of which eight were included in the final analysis (Fig. 1). Out of the eight studies included, six were clinical trial papers [1–6], and two were real-world studies [7, 8]. Out of the twenty-six papers excluded, seventeen were real-world studies not on ibrutinib dosage or discontinuation, two were study protocols, six were molecular/pharmacokinetic studies, and one was a phase 1 study.

To identify ibrutinib real-world studies in PubMed, the key title MeSH terms used were “Ibrutinib” and “real-world” sorted by best match and filtered by studies published in the last 5 years. Nine papers were identified. To broaden our search, the key non-title MeSH terms used were “Ibrutinib and low or lower and dose or dosage” and “chronic lymphocytic leukemia or CLL” sorted by best match filtered by studies published in the last 5 years. This identified fifty studies. Combining both searches, we therefore identified a total of fifty-nine studies (Fig. 1). Five papers were included in the final analysis which included one clinical trial and four real-world studies [9–13]. Out of the fifty-four studies excluded, thirty-nine were not relevant to ibrutinib dosage, four studies had been previously identified, two were pharmacokinetic studies, seven were phase I clinical trials, and two were not relevant to CLL (Fig. 1).

To identify ibrutinib clinical trial and “real-world practice” studies in Scopus, the key title MeSh terms used were “Ibrutinib”, “low or lower”, “dose or dosage”, and “chronic lymphocytic leukemia or CLL”. A total of thirty-three studies of which one “real-world practice” was included in the final analysis [14]. Out of the thirty-two studies excluded, twenty-five were not relevant to ibrutinib dosage, five had previously been identified, and two were not relevant to CLL (Fig. 1).

Additional supplementary searching, including consulting cited references, identified eight additional “real-world practice” studies [15–22] and seven clinical trial papers [23–29]. In summary, we identified twenty-nine studies including fourteen papers reporting on clinical trials (eight original papers, five papers reflecting longer-term follow-up of a retrospective trial and one sub-analysis of the RESONATE trial [2]) and fifteen “real-world” studies providing information on dose modification practice, rationale, and clinical sequelae in the context of routine clinical care (Fig. 1).

## Results

### Clinical characteristics: clinical trial participants vs patients in real-world practice

Baseline patient characteristics, including age, performance status, and chromosomal abnormalities, of patients treated in 9 respective clinical trials vs those of patients treated in the “real-world practice” setting are shown in Tables 1 and 2, respectively [1, 3–11, 13–17, 24–27]. In both types of studies, ibrutinib was mainly used to treat CLL. Patients with CLL ranged from 90–100% in clinical trials vs 89–100% in “real-world practice” studies. However, in studies including both patients with treatment-naïve CLL and relapsed disease, real-world studies reported a higher proportion of patients with relapsed disease compared to the proportion of patients with relapsed disease in clinical trial studies; 89.9% (range 81–97.4%) vs 62.1% (range 38.4-77%), respectively. Ibrutinib was more often used in patients with relapsed disease in these “real-world” practice studies. Furthermore, ibrutinib was more often used in patients with poor performance status (ECOG ≥ 2) in real-world studies. The majority of “real-world” practice studies included roughly a third of patients with poor performance status (ECOG ≥ 2), with the exception of the report by Akhtar et al., in which a lower proportion of patients with ECOG ≥ 2 (7%) were included. In all the clinical trials, patients with a poor performance status, as measured by ECOG ≥ 2, contributed to a very small proportion of the total number of study patients (0–3%), with the exception of the RESONATE-2 study, which included a slightly higher proportion of patients with ECOG ≥ 2 (8%).

**Table 1:** Patient characteristics in clinical trials

1st author	Study	Number of patients	Median age (range)	Therapy (%)		Diagnosis		del 11q22.3 (%)	del 17p13.1 (%)	CYP3A45 strong inhibitors
				Front-line	Relapse	CLL (%)	SLL (%)			
Byrd et al.	RESONATE	195	67 (30–86)	0	100	95	5	32	32	Excluded
Burger et al. [3]	RESONATE-2	136	73 (65–89)	100	0	90	10	21	0	Excluded
O’Brien et al. [5]	RESONATE-17	144	64 (57–72)	0	100	95	5	16	100	Excluded
Ahn et al. [6]	NCT01500733	86	66 (33–85)	61.6	38.4	1	0	NR	58	NR
Byrd et al. [25]	PCYC 1102 phase 1b/2 (NEJM 2013)	85	68 (37–82)	0	100	96	4	36	33	NR
O’Brien et al. [24]	PCYC 1102 phase 1b/2 (Lancet 2014)	31	71 (65–84)	100	0	94	6	3	6	NR
Byrd et al. [26]	PCYC 1103 3-year FU (Blood 2015)	132	68 (37–84)	23	77	96	4	27	27	Avoided
Coutre et al. [4]	PCYC 1103 44-month FU	94	68 (37–84)	29	71	NR	NR	23	27	Avoided
Chen et al. [27]	NCT02801578	11	68 (52–79)	NR	NR	100	0	0	0	NR

**Table 2:** Patient characteristics in “real-world practice” studies

1st author	Number of patients	Median age (range)	Therapy (%)		Diagnosis		del 11q22.3 (%)	del 17p13.1 (%)	CYP3A45 strong inhibitors
			Front-line	Relapse	CLL (%)	SLL (%)			
Mato et al. [8]	197	66 (NR)	19	81	100	0	NR	NR	Excluded
Ysebaert et al. [10] (French cohort)	428	70 (33–93)	2.6	97.4	100	0	NR	45.1	NR
Winqvist et al. [11] (Swedish cohort)	95	69 (42–86)	0	100	98	2	18	63	Excluded
Akhtar et al. [16]	70	68.0 (48.4–92.0)	10	90	100	0	24	10	NR
Mato et al. [13]	391	68 (36–96)	100	0	100	0	17.1	29.8	NR
Finnes et al. [14]	118	59 (29–83)	5.9	94.1	89	11	13	17	Included
UK CLL forum [7]	315	69 (42–93)	0	100	100	0	NR	28.3	NR
Mato et al. [9]	616	60 (22–95)	13	87	100	0	35	26	NR
Iskierka-Jażdżewska et al. [17] (Polish cohort)	165	63 (40–84	0	100	97	3	NR	18.4	Excluded
Mato et al. [15]	178	60 (33–89)	100	0	100	0	37	37	Excluded

Patients with adverse cytogenetics consisting of del 11q22.3 or del 17p13.1 were variably reported in both clinical trial and real-world studies. The average proportion of people with 11q deletions was higher in real-world studies compared to the average proportion of people with 11q deletions in clinical trials (24% vs 19.75%). In 5/9 clinical trial studies and in 6/10 real-world studies, about a third (13–37%) of CLL patients had del 11q22.3. The percentage of patients with del 17p13.1 in clinical trials can be summarized as follows: (1) 100% in the RESONATE-17 study ( they only included patients with del 17p13.1), (2) 58% in 1/9 studies [6], (3) 27–33% in 4/9 studies [1, 4, 25, 26], (4) 6% of patients the PCYC 1102 study [24], and (5) 0% in RESONATE-2 due to exclusion and 0% in NCT02801578 study due to chance. All real-world studies included patients with del 17p13.1 except Mato et al. which did not report. The percentage of patients with del 17p13.1 ranged from as few as 10% [16] to as much as 45–63% [10, 11] with the majority of studies (6/10), including roughly one third (17–37%) of patients who possessed this adverse prognostic feature [7, 9, 13–15, 17]. Overall, real-world studies had a higher proportion of people with 11q and 17p deletions. Finally, only one real-world study clearly reported the use of ibrutinib in patients on strong CYP3A4 inhibitors while all RESONATE trials excluded concomitant use of strong CYP3A4 and many studies did not report on their inclusion or exclusion. Overall, “real-world practice” studies reflected the use of ibrutinib in younger patients, with more adverse performance status and prognostic factors, as evidenced by a higher proportion of people with relapsed CLL, ECOG ≥ 2, and with 11q and 17p deletions, and included patients on strong as well as moderate or weak CYP3A4 inhibitors and or inducers.

### Discontinuation of ibrutinib

#### Clinical trials

Discontinuation rates in the setting of formally conducted clinical trials are shown in Table 3 [1, 2, 3, 4, 5, 6, 23, 24, 25, 26, 28]. The average discontinuation rate was 32% (range 12.5–66%). In 5/11 studies, CLL progression accounted for about half of the discontinuation rates, ranging from 40 to 54%. In RESONATE-2, toxicity accounted for 64.7% of discontinuations, in the longer-term follow-up report of RESONATE-2 (28.5 months), toxicity, combined with death due to other causes, accounted for 78.6% of discontinuation. In the remaining 9 studies, adverse events accounted for roughly one third of the discontinuations, ranging from 22–40% [1, 4, 5, 6, 23, 24, 25, 26, 28].

**Table 3:** Discontinuation rates of ibrutinib in published clinical trials

1st author	Study	Median duration of treatment in months (range)	Discontinuation rate (%)	Reason for dose discontinuation (%)				Dose reduction 1 year (%)
				Non-relapse disease			Relapsed disease	
				Toxicity/AE	Death not secondary to CLL/AE	Patient preference/other	CLL progression	
Byrd et al. [1]	RESONATE	8.6 (0.2–16.1)	13.8	29.6	29.6	7.4	33.3	NR
Byrd et al. [1]	RESONATE long-term FU	41 (0.2–50.1)	53.8	22	11.4	16.1	50.5	6
Burger et al. [3]	RESONATE-2	17.4 (0.7–24.7)	12.5	64.7	17.6	5.9	11.8	NR
Barr et al.	RESONATE-2 long term FU	28.5 (0.7–35.9)	20	78.6		7.1	14.3	9
O’Brien et al. [23]	RESONATE-17	11.5 (11.1–13.8)	50	33		20	47	7
Ahn et al. [6]	Ahn et al.	4.8 years (4–6 years)	43	13	11	22	54	NR
Byrd et al. [1]	NEJM 2013-PCYC 1102	20.9 (0.7–26.7)	36	22.5	0	42	35.5	NR
O’Brien et al. [24]	Lancet 2014-PCYC 1102 phase 1b/2	22.1 (18.4–23.2)	16	40	NR	40	20	NR
Byrd et al. [1]	PCYC 1103	25 (0.3–45)	40	28	NR	30	42	NR
Coutre et al. [4]	PCYC 1103	30 (1–44)	34	37	0	25	28.1	NR
O’Brien et al.	PCYC 1103	62 (1–75)	66	31	NR	27.6	40	NR

In a retrospective study, Jain et al. reported reasons for discontinuation of ibrutinib in patients with CLL from 7 distinct clinical trials conducted between 2010 and 2015 [18]. The study was not included in Table 1 because the pooled analysis from 7 different clinical trials made it difficult to attribute specific patient characteristics to the individual regimens utilized. However, with 320 patients, it is the largest study to report on the rates and reasons for ibrutinib discontinuation. The discontinuation rate of 32% was similar to the 32% average discontinuation rate for the trials included in Table 3, as detailed above. Out of the patients who discontinued ibrutinib, 52% received ibrutinib monotherapy, 34% received ibrutinib with rituximab, and 14% received ibrutinib with bendamustine and rituximab. CLL progression accounted for 21% of ibrutinib discontinuations, lower than the average of 34% noted for the 11 clinical trials evaluated. The most common reason for discontinuation in this study was intolerance/toxicity (32%) followed closely by other adverse events (31%), respectively.

Similarly, a study by Maddocks et al. was not included in Tables 1 and 3, because it was a pooled analysis of discontinuation rates derived from 4 different clinical trial participants treated with ibrutinib monotherapy or ibrutinib with ofatumumab [19]. The investigators reported a similar rate of discontinuation (25%), similar rates of toxicity/other adverse events leading to discontinuations (59%), and similar rate of CLL progression (17%) to the rates found in the Jain et al. study. In addition, 23% of patients discontinued ibrutinib due to Richter’s transformation.

In the RESONATE long-term follow-up [28], RESONATE-2 long-term follow-up [2], and PCYC-1103 5-year follow-up [23], the prevalence of adverse events leading to dose reduction was analyzed over time. The RESONATE long-term follow-up showed that the prevalence of adverse events leading to dose reduction remained consistent over time (6%, 9%, 4%, and 7% over years 0 to 1, 1 to 2, 2 to 3, and 3 to 4, respectively). The RESONATE-2 long-term follow-up and PCYC-1103 5-year follow-up showed that dose discontinuations and dose reductions resulting from adverse events occurred more frequently during the first year and tended to decrease over time (9% within 1st year, 5% within 2nd year, 4% after 2nd year ) on ibrutinib monotherapy [23, 28].

#### “Real-world practice” studies

Discontinuation rates reported in real-world studies are shown in Table 4 [7, 9–11, 13–17]. The average discontinuation rate was 33.5% (range14.5–43%). CLL progression accounted for almost half of the discontinuation rates observed in one study [11]. In 5/9 studies, adverse events accounted for over half of the discontinuations ranging from 50.2–63.1% [7, 9, 13–15].

**Table 4:** Discontinuation rates of ibrutinib in “real-world practice” studies

1st author	Median duration of treatment (months)	Discontinuation rate	Reason for dose discontinuation					Dose reduction over 1 year (%)
			Non-relapse disease			Relapsed disease		
			Toxicity/AE	Death not secondary to CLL/AE	Patient preference/other	CLL progression	CLL transformation	
Ysebaert et al. [10] (French cohort)	3 (1–10)	14.5	37	35	13	15		NR
Winqvist et al. [11] (Swedish cohort)	27 (0.6–38)	24	43	NR	14	43		22
Akhtar et al. [16]	21.9	40	25	NR	22	32	21	31.3
Mato et al. [9]	13.8 (1–76)	24	60	3.2	14.8	12.8	9.6	17.4
Finnes et al. [14]	13	20.3	58	NR	NR	25	16.7	21.2
UK CLL forum [7]	16	26.3	55	3.6	7.2	16.9	16.9	26
Mato et al. [9]	17 (1–60)	24, 43	63.1, 50.2	5.3, 12.1	10.5, 6.7	15.8, 20.9	5.3, 4.6	15, 20
Iskierka-Jażdżewska et al. [17] (Polish Cohort)	9.5 (0.1–22.2)	19.4	9.7	NR	2.4	7.3		NR
Mato et al. [15]	5 (0.25–41)	100	51	NR	13	28	8	11

William et al. analyzed the reasons for ibrutinib discontinuation in patients with non-Hodgkin lymphoma (NHL) and CLL [12]. We did not include this study in Table 2 given the more diverse patient population with reported data combining both CLL and NHL patients. However, patients with CLL represented the majority of subjects included in this large retrospective cohort, with 115 out of the 170 patients diagnosed with CLL. In this study, the discontinuation rate (30%) for the entire patient population as a whole was comparable to the rate reported in our 8 other real-world studies which included only patients with CLL. Disease progression (33%) and adverse events (37.2%) as reasons for discontinuation were lower in this study respectively than that observed for other real-world CLL only studies, as detailed above. The actual number of CLL patients that contributed to these rates of discontinuation is unfortunately not provided. Overall, the reasons for ibrutinib discontinuations differed between patients in clinical trials vs real-world studies with more patients discontinuing ibrutinib due to adverse events in real-world studies and more patients discontinuing ibrutinib due to progression of disease in clinical trials.

### Ibrutinib dosing in CLL (clinical trial and clinical practice setting) post allogeneic stem cell transplant

Little is known about the use of ibrutinib in patients with relapsed CLL following allogeneic hematopoietic cell transplant (HCT). Ryan et al. reported on the tolerability of ibrutinib in 27 patients with relapsed CLL ollowing allogeneic transplantation [20]. Sixteen patients, treated in the context of 4 separate multi-centered trials, and an additional 11 patients treated at Stanford University as part of routine care were included in the analysis. All patients received 420 mg of ibrutinib daily, except for 2 patients in the clinical trial group who received 840 mg daily. For the 16 patients treated on clinical trials, the median duration of ibrutinib therapy was 19 months (range 0.4–39 months) with 14 patients treated for longer than 12 months. At the time of data cutoff, the ibrutinib discontinuation was 31%. Reasons for discontinuation included adverse events (12.5%), progression (12.5%), and withdrawal of consent (1). In the 11 patients treated at Stanford University post-transplant as part of routine clinical practice, the median duration of ibrutinib treatment of 8.4 months (range 0.5–21.4) with 45% of patients discontinuing therapy at the time of this analysis. No dose adjustments were noted in either the clinical trial or clinical practice setting.

#### Discontinuation of ibrutinib: summary of clinical trials vs real-world studies

In all clinical trials, patients received oral ibrutinib (at a dose of 420 mg once daily) until disease progression or the occurrence of adverse events. Adverse events led to dose reductions during the 1st year of treatment in 5–10% of patients (average 7%). In real-world studies, adverse events led to dose reductions during the 1st year of treatment in a slightly larger group of individuals (11–31.3%).

### Dose reduction of ibrutinib

A low incidence (6–9%) of dose reductions after being on treatment for more than a year was observed in 3/9 clinical trials [5, 28, 29]. In the long-term follow-up study of patients enrolled on the RESONATE-2, the prevalence of dose reductions appeared to decrease over time with a median follow-up of 28.5 months. In all clinical trials, patients received oral ibrutinib (at a dose of 420 mg once daily) until disease progression or the occurrence of adverse events. Adverse events led to dose reductions during the 1st year of treatment in 5–10% of patients (average 7%).

Dose reductions over the first year of therapy were observed more frequently (11–31.3%) in 7/9 “real-world practice” studies [7, 8, 11, 13, 14, 15, 16]. With a median follow-up of 27 months comparable to the median follow-up of the long-term RESONATE-2 study, Winqvist et al. reported 22% of patients whose ibrutinib dose was reduced over their first year over therapy compared to 9% of patients in the long-term RESONATE-2 study. In one study, a higher proportion of dose reductions were noted to occur earlier (within 3 months of treatment initiation) [16].

One “real-world practice” study reported that the prevalence of dose reductions decreased over time with a median follow-up of 21.9 months, comparable to that reported for the RESONATE-2 clinical trial [16].The most common reasons for ibrutinib dose reduction, in two of the real-world studies, were cytopenias and infection [11, 16].

### Impact of ibrutinib dose adjustments

Barr et al. performed a retrospective analysis of the clinical impact of ibrutinib dose adherence in 195 patients from the RESONATE trial [2]. In this trial, patients whose course of therapy deviated little from the planned regimen, and who adhered closely to the prescribed regimen, referred to as planned dose intensity (*n* = 155 patients) were reported to have an improved PFS as compared to those individuals who required either prolonged discontinuation of ibrutinib or for whom the planned dose intensity was not maintained (*n* = 38 patients) due largely to adverse events and prolonged toxicity. Although not statistically significant, these small retrospectively compared groups differed with regard to Rai stage of disease, number of prior therapies, and creatinine clearance. Despite differences in renal function, no comparison in the pharmacokinetics of ibrutinib was provided between the two groups. Since ibrutinib is cleared by the kidney, one might reasonably postulate that in patients receiving the planned dose of ibrutinib, the area under the curve would be greater for patients with impaired renal function as compared to those with normal renal function, enhancing their predisposition to untoward toxicity. This in turn would make it difficult for patients to receive therapy, leading to a worse progression free survival (PFS) as opposed to the reduced dose in and of itself. Unfortunately, in this study, no information was provided for AUC as a function of creatinine clearance. The potential for the introduction of this type of confounder, given the hypothesis generating analysis, makes it difficult to know whether the prolonged need to withhold therapy due to unacceptable toxicity is the causative factor as opposed to the lack of maintaining dose intensity, as a plausible explanation. This in turn suggests that the avoidance of toxicity in the first place, through the use of an appropriately adjusted dose based on renal function, to achieve the desired area under the curve might have enabled patients to have an equally good PFS. This hypothesis is supported by the reported observation that eleven of twenty-six patients who restarted ibrutinib after developing progressive disease while having their dose of ibrutinib held were without clinical progression for a period of time (> 6.5 months). In addition, investigators also found that patients missing ≥ 8 consecutive days of ibrutinib had a shorter median PFS vs those missing < 8 days (10.9 months vs not reached).

Retrospective observational studies conducted in the context of “real-world practice” have attempted to address the impact of dose modifications on outcome with less success, given the inherent potential for selection bias and the introduction of confounders. A “real-world practice” study conducted by UK CLL Forum attempted to examine the role of ibrutinib dosing in routine clinical practice on progression free survival and overall survival [7]. In this study, clinicians were provided the opportunity to contribute anonymized data through an established database. Data from three hundred and fifteen patients from 63 medical centers across the UK was collected. Data for all parameters assessed was not available on all patients; however, the median number of prior therapies was 2, with 83.5% of patients having FISH + for 17pdeletion, consistent with a higher risk group of patients. In this cohort, 83 patients discontinued therapy at the end of 1 year, predominantly for progression of disease, resulting in a poor overall survival, as anticipated. To better delineate the role of dose modifications within the first year of treatment, the investigators divided their cohort into three respective groups: group A who received standard dose ibrutinib with no dose reductions and no treatment breaks of > 14 days, group B who received any dose reduction but no treatment breaks of > 14 days, and group C who had ibrutinib therapy interrupted for > 14 days with or without dose modifications. Median follow-up was 16 months. No difference in disease free survival (DFS) or overall survival (OS) was observed between groups A and B, suggesting that dose modification of ibrutinib in and of itself did not portend an adverse outcome; however, 42/92 patients in group C, who were identified to have had ibrutinib withheld for > 14 days, had a much poorer DFS and OS, suggesting that this modification in dosing, related to the inability to deliver the needed treatment, is associated with a poor outcome in this context. To further delineate the impact of dosing on outcome, the investigators conducted a post 1-year analysis of all patients in group A, B, and C, respectively. In this prospective analysis, patients who had dose reductions in the first year (group B) had no difference in DFS and OS from patients who received the standard regimen (group A). Furthermore, post 1-year survival did not appear to be affected by dose reductions (C1) but was compromised by temporary and permanent breaks in therapy. These data suggest that other factors, as yet to be determined, related to the need to stop ibrutinib therapy are of greater impact on outcome than modifications of ibrutinib dosing per se.

In a second retrospective study, William et. al. attempted to evaluate the impact of ibrutinib dose reductions and interruptions on outcomes in patients with CLL (51% high risk) and NHL [12]. In the CLL cohort, 29% of patients had a reduction in dose, and 57.6% had at least one transient dose interruption (of any length) over the study period. Median follow-up time was 14.28 months (range 0.36–65.77 months). These investigators attempted to assess the impact of dose adherence within the first 8 weeks of treatment, defining a dose adherence to the expected treatment regimen of more or less than 80%. Taking this approach, the investigators reported that CLL patients, who had a dose adherence to the standard regimen of ibrutinib of < 80% within that 8-week period, experienced a worse PFS; however, the number of patients this reflects out of the 115 CLL patients and their respective characteristics or reasons for non-adherence, included in the analysis is not provided, making this difficult to discern the real impact of dose modifications itself as opposed to being a marker of other adverse features, leading to poorer outcomes.

In a third retrospective study, Mato et al. attempted to evaluate the impact of age and del 17p13.1 on dose reductions in patients with CLL [13, 15]. This study included patients < 65 years old and with del 17p13.1 who had been excluded from RESONATE-2 trial. Median follow-up was 13.8 months (range 1–76 months). Age was associated with greater likelihood of initial and on treatment ibrutinib dose reduction with older patients more likely to start at a dose below 420 mg or to have their dose reduced during treatment to achieve steady state. The presence of del 17p13.1, however, did not affect the starting dose or on treatment dose reductions. In total, about 7% of patients required an initial reduced dose, and 17% required a dose reduction during treatment. As per the Williams study, investigators attempted to evaluate the impact of ibrutinib dose reduction on PFS. They reported a worse 12-month PFS in patients receiving a reduced dose of ibrutinib, although the reason for reducing the dose was not reported. Patients comorbidity index, prior treatments, renal function, and other inherent patient characteristics could have contributed to patients requiring a dose reduction leading to a worse outcome. Given these numerous possible cofounders, the association reported does not establish a causal relationship between reduced ibrutinib dose and PFS.

Finally, a pilot study by Chen et al. attempted to evaluate the impact of reductions in ibrutinib dosing on Bruton tyrosine kinase (BTK) levels and expression [27]. Eleven patients received 420 mg/day in cycle 1280 mg/day in cycle 2, and 140 mg/day in cycle 3. In this study, the investigators demonstrated that total BTK protein decreased over the course of the 3 cycles, leading to a similar BTK occupancy with a reduced dose of ibrutinib as well as comparable pharmacodynamic and biological properties. Since BTK occupancy and biological activity is preserved at lower doses of ibrutinib, this study suggests that alterations in treatments through dose interruptions and reductions are likely not the root cause for worse clinical outcomes in patients.

### Impact of CYP34A inducers/inhibitors on ibrutinib dosing

Ibrutinib is extensively metabolized and eliminated by Cytochrome P450 3A (CYP3A). For this reason, the concomitant use of ibrutinib and CYP3A inhibitor and inducers could potentially promote enhanced ibrutinib toxicity or reduced efficacy, respectively. Because of the potential for adverse effects due to drug interactions, the concomitant use of strong CYP3A inhibitors was not permitted in the RESONATE and RESONATE-2 trials; however, mild and moderate CYP3A inhibitors were permitted. CLL more commonly affects older adults who often have comorbid conditions, requiring the use of medications that alter CYP3A metabolism. In addition, patients undergoing hematopoietic stem cell transplantation (HSCT) are often treated with complex medical regimens including CYP 3A4 inhibitors/substrates which may impact the bioavailability of ibrutinib. To this end, Finnes et al. evaluated concomitant medication use in 118 ibrutinib-treated CLL patients, in the clinical practice setting at the Mayo Clinic [14]. Ninety percent of this patient cohort had relapsed/refractory CLL (90%) with a median age of 59 (range 29–83). In anticipation of starting ibrutinib 21/118 CLL, patients (16%) were found to be on treatment with either a moderate or strong CYP3A inhibitor or inducer. As a result, prior to the initiation of ibrutinib, patients had either an adjustment in their concomitant medication to avoid drug interactions [5] or had dose modifications based on predicted changes in and preservation of desired pharmacokinetic properties associated with ibrutinib therapeutic responses. To achieve ideal pharmacokinetics while either remaining on or requiring the addition of concomitant medications known to alter CYP3A functional activity, the following adjustments in ibrutinib dose were made as follows: 140 mg once every other day (strong CYP3A inhibitors) and 140 mg once a day (moderate CYP3A inhibitors). In this study, based upon dosing to achieve desired pharmacokinetic properties, no difference in discontinuation rates of ibrutinib at 12 months or in the 18-month PFS between patients on medications known to impact CYP3A activity versus those who did not was observed. In addition, no difference in time to ibrutinib discontinuation was noted [14].

To better predict appropriate dosing recommendations for patients receiving concomitant moderate to strong CYP3A inhibitors, Zwart and colleagues developed a physiologically based pharmacokinetic model (PBPK) [22]. Using ketoconazole as a strong CYP3A inhibitor and rifampin as a strong CYP3A inducer prototypes respectively, this population-based simulation PBPK model, additionally validate by clinically observable data (healthy volunteers), was able to predict the impact of specific drug-drug interactions leading to US, Canadian, and European regulatory agency dosing recommendations for ibrutinib in the context of drugs that impact CYP3A functional activity. These recommendations include the avoidance of strong and moderate CYP3A inhibitors whenever possible, when using ibrutinib or, if unavoidable, lowering the dose of ibrutinib to 140 mg po daily. Similarly, based on this study, the use of strong CYP3A inhibitors was discouraged, due to the likelihood of rendering ibrutinib ineffective. To confirm these established dosing recommendations and to shed additional light on the potential for drug-drug interactions between ibrutinib and moderate to strong CYP3A inhibitors, de Jong et. al. studied the impact of erythromycin and voriconazole and moderate and strong CYP3A inhibitor prototypes respectively, on the pharmacokinetics of 140 mg of ibrutinib, in the context of a multicenter phase I study in 26 patients with low grade B cell malignancies including 14 patients with CLL (53.8%) [21]. This study demonstrated that ibrutinib 140 mg administered in combination with voriconazole or erythromycin provided pharmacological levels of drug comparable to that observed for patients treated with 560 mg of ibrutinib alone, doses routinely used in patients with marginal zone lymphoma, and higher than the exposure usually observed with the standard dose of ibrutinib (420 mg daily), utilized in patients with CLL, suggesting room for further reduction in ibrutinib dosing, to achieve comparable biologically and clinically effective levels.

These studies collectively suggest that adjustment of ibrutinib dosing is warranted in the context of the need for co-administration of moderate to strong CYP3A inhibitors for comorbid conditions, to achieve pharmacologically comparable therapeutic levels of ibrutinib, thereby maintaining disease specific treatment efficacy while avoiding the potential for added toxicity that standard dosing would likely ensue.

## Discussion

From our review of both clinical trials and real-world practice studies to date, we can conclude that patients receiving strong or moderate CYP3A inhibitors, defined by the US, Canadian, and European regulatory agency, should be treated with 140 mg or 280 mg of ibrutinib respectively [22]. Despite differences in patient characteristics, the average discontinuation rate of ibrutinib was similar between clinical trials and “real-world practice” as follows: 32% (range 12.5–66%) vs 33.5% (range 14.5–43%), respectively. However, the reason for discontinuation differed with toxicity being a more common reason for discontinuation in “real-world practice” compared to clinical trials [1–4, 6, 7, 9–11, 13–17, 23–26, 28]. In the largest real-world study including 616 patients with CLL treated with ibrutinib, the three most common toxicities were arthralgia (41.6%), atrial fibrillation (25%), and rash (16.7%) [9]. More patients had adverse events leading to ibrutinib dose reductions in “real-world practice” studies compared to clinical trial studies (7% vs 24%). The most common adverse events causing ibrutinib dose reductions were infection and cytopenia in two real-world studies [11, 16]. In addition, we can hypothesize, based on differences in performance status as a surrogate, that real-world patients likely suffered from additional comorbidities, in particular abnormal liver function, normally excluded in the context of rigorous clinical trials, possibly contributing to the increase in side effects observed. Unfortunately, this has not been systematically reported to enable one to clearly prove this hypothesis. Both clinical trials and real-world studies showed that the prevalence of dose reductions decreased over time. This is possibly due to the fact that the majority of adverse events occur earlier on in treatment [2, 6]. The key differences in ibrutinib dosing between clinical trials and “real-world practice” studies are summarized in Table 5.

**Table 5:** Summary of ibrutinib dose discontinuation rates or modification in clinical trials vs “real-world practice” studies

	Clinical trial	Real-world
**Average discontinuation rate due to toxicity**	36.3% (range 13–64.7%)	45.2% (range 9.7–63.1%)
**Average discontinuation rate due to relapsed disease**	34.2% (range 11.8–50.5%)	25.5% (range 12.8–53%)
**Average dose reduction over 1 year**	7.3% (range 6–9%)	20.5% (range 11–31.3%)

Given the current recommended guidelines to reduce ibrutinib dosing, when patients require the concomitant use of a moderate or strong CYP3A inhibitor, it is tempting to speculate on the potential cost savings that could be realized, if patients receiving ibrutinib therapy were to be placed routinely on an inexpensive and otherwise well tolerated CYP3A inhibitor. However, the safety of this approach, the long-term tolerability, and the clinical efficacy of ibrutinib therapy in this context has not been formally evaluated. The conduct of a well-designed prospective clinical trial, to evaluate this possible therapeutic strategy, will be needed before such an approach could be considered in clinical practice.

The clinical impact of dose modifications remains unclear. Patients who are not able to maintain the dose due to either prolonged dose reductions or interruptions in treatment appear to have worse outcomes though it remains unclear whether it is due to the patient’s inability to receive the standard dose, or whether it results from the dose modification itself [2, 7, 12]. At the current time, best practice dosing recommendations include reduction in the ibrutinib dose for patients receiving moderate or strong CYP3A inhibitors, as detailed above and when treating patients with liver disease comorbidity [30]. Based upon this review, these dosing recommendations as well as clinical judgement, should guide dosing parameters in an effort to optimally balance clinical therapeutic efficacy and safety.

Some studies have shown worse outcomes in patients requiring dose reductions while others have shown no decrease in treatment efficacy [7, 12, 13, 15]. These controversial findings highlight the numerous confounders that potentially impact ibrutinib dose modifications and outcomes, rendering it difficult to draw any definitive dosing recommendations at this time.

## Conclusion

The impact of ibrutinib dose modifications on clinical outcome remains unclear. Patients on concomitant CYP3A inhibitors should be prescribed a lower dose than the standard 420 mg daily, in order to maintain comparable pharmacologic properties. Modification of ibrutinib doses in real-world practice reflects an increase in side effects observed as compared to that reported in clinical trials. This likely reflects differences in patient characteristics and comorbidities among real-world patients as opposed to those enrolled onto clinical trials. Further research is required to establish definitive clinical practice guidelines.

## Data Availability

Not applicable

## Abbreviations

BTK: Bruton’s tyrosine kinase
CLL: Chronic lymphocytic leukemia
PBPK: Physiologically based pharmacokinetic
FISH: Fluorescence in-situ hybridization
CYP: Cytochrome P450
ECOG: Eastern Cooperative Oncology Group
MeSH: Medical subject headings
